# The Many Facets of Erythropoietin Physiologic and Metabolic Response

**DOI:** 10.3389/fphys.2019.01534

**Published:** 2020-01-17

**Authors:** Sukanya Suresh, Praveen Kumar Rajvanshi, Constance T. Noguchi

**Affiliations:** Molecular Medicine Branch, National Institute of Diabetes and Digestive and Kidney Diseases, National Institutes of Health, Bethesda, MD, United States

**Keywords:** erythropoietin, erythropoietin receptor, nitric oxide, gender-specific, obesity, inflammation, bone

## Abstract

In mammals, erythropoietin (EPO), produced in the kidney, is essential for bone marrow erythropoiesis, and hypoxia induction of EPO production provides for the important erythropoietic response to ischemic stress, such as during blood loss and at high altitude. Erythropoietin acts by binding to its cell surface receptor which is expressed at the highest level on erythroid progenitor cells to promote cell survival, proliferation, and differentiation in production of mature red blood cells. In addition to bone marrow erythropoiesis, EPO causes multi-tissue responses associated with erythropoietin receptor (EPOR) expression in non-erythroid cells such neural cells, endothelial cells, and skeletal muscle myoblasts. Animal and cell models of ischemic stress have been useful in elucidating the potential benefit of EPO affecting maintenance and repair of several non-hematopoietic organs including brain, heart and skeletal muscle. Metabolic and glucose homeostasis are affected by endogenous EPO and erythropoietin administration affect, in part via EPOR expression in white adipose tissue. In diet-induced obese mice, EPO is protective for white adipose tissue inflammation and gives rise to a gender specific response in weight control associated with white fat mass accumulation. Erythropoietin regulation of fat mass is masked in female mice due to estrogen production. EPOR is also expressed in bone marrow stromal cells (BMSC) and EPO administration in mice results in reduced bone independent of the increase in hematocrit. Concomitant reduction in bone marrow adipocytes and bone morphogenic protein suggests that high EPO inhibits adipogenesis and osteogenesis. These multi-tissue responses underscore the pleiotropic potential of the EPO response and may contribute to various physiological manifestations accompanying anemia or ischemic response and pharmacological uses of EPO.

## Introduction

**FIGURE 1:**
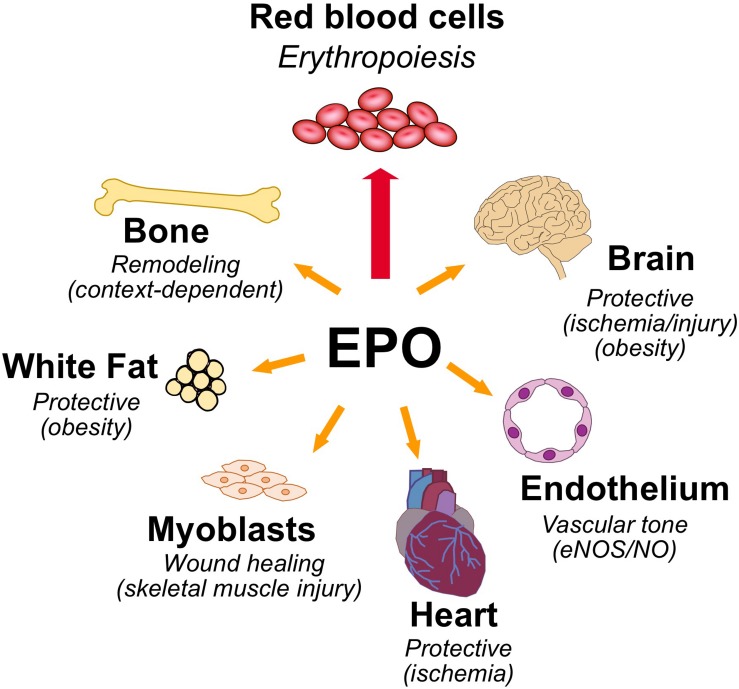
Pleiotropic effects of erythropoietin. High level of EPOR on erythroid progenitor cells accounts for the sensitive erythropoietic response in the bone marrow to hypoxic induction of EPO. EPOR expression determines EPO response and expression beyond erythroid tissue provides for EPO response in non-hematopoietic tissues that include the following: brain for a neuroprotective and metabolic response; cardiovascular system for regulating vascular tone and oxygen delivery in endothelium and protection in heart against ischemic injury; skeletal muscle for muscle maintenance and repair; white adipose tissue for protection for inflammation associated with diet-induced obesity and fat mass accumulation, particularly in males; and bone remodeling.

Erythropoietin is the hormone that regulates the daily production of 200 billion new red blood cells in the human body. Red blood cells are a renewable resource with a limited lifespan of about 120 days, have an intracellular protein content of about 95% hemoglobin, a tetrameric globular protein that binds oxygen cooperatively, and function primarily to transport oxygen from the lungs to the tissues. EPO binding to erythroid progenitor cells promotes their survival, proliferation, and differentiation to mature erythrocytes. EPO production is hypoxia inducible and is made in the interstitial cells in the adult kidney (Kobayashi et al., 2017; Anusornvongchai et al., 2018). In response to anemia, ischemic stress or high altitude, EPO production is induced (Pugh and Ratcliffe, 2017) and stimulates erythroid progenitor cells in the bone marrow to expand the erythroid lineage thus markedly increasing erythropoiesis and mature red blood cell production. EPO and EPOR on the surface of erythroid progenitor cells are required for red blood cell production and mice with targeted deletion of EPO or EPOR die during embryonic development of severe anemia (Wu et al., 1995; Lin et al., 1996). However, EPOR expression is not restricted to erythroid tissue. This review will address EPO activity in erythroid cells and in select non-hematopoietic tissues expressing EPOR and lessons gleaned from studies in animal models on the protective effects of EPO in ischemic injury and wound healing, regulation of metabolic homeostasis including gender specific EPO response and bone remodeling (Figure 1).

## Erythropoietin Action in Erythroid Cells

Erythropoietin is a glycoprotein produced primarily in the fetal liver and adult kidney to regulate red blood cell production. Human EPO is encoded as a 193 amino acid polypeptide with a NH_2_-terminal 27 amino acid signal peptide and three potential N-linked glycosylation sites; posttranslational cleavage of the carboxyl terminal arginine gives rise to a 165 amino acid mature polypeptide for human EPO (Jacobs et al., 1985; Lin et al., 1985; Recny et al., 1987). Recombinant EPO produced in Chinese hamster ovary cells yields a glycoprotein with apparent molecular weight about 34,000 that is biologically active (Recny et al., 1987). EPO received approval in 1989 from the U.S. Food and Drug Administration for clinical treatment of anemia associated with chronic renal failure due to insufficient EPO production, which has markedly improved treatment of this disease (Paoletti and Cannella, 2006; Jelkmann, 2013; Wright et al., 2015).

### Sites of EPO Production

During mammalian development, observations of EPO production in mice suggest that EPO is first expressed transiently in neural crest cells during mid-gestation to stimulate yolk sac primitive erythropoiesis for oxygen transport in mid-stage embryos (Malik et al., 2013; Suzuki et al., 2013; Hirano and Suzuki, 2019). As development progresses, the liver becomes the site of EPO production and definitive erythropoiesis (Palis, 2014; Palis and Koniski, 2018). EPO is required for definitive erythropoiesis and knockout of EPO or EPOR in mice results in death *in utero* around day 13.5 due to disruption of erythropoiesis in the fetal liver resulting in severe anemia (Wu et al., 1995; Lin et al., 1996). By the last third of gestation in mammalian development, the site of EPO production gradually switches to the kidney which becomes the major site of EPO production in the adult (Zanjani et al., 1981; Dame et al., 1998) and red blood cell production switches from the fetal liver to bone marrow, the site of adult hematopoiesis (Ho et al., 2015). Interstitial peritubular cells of the kidney are the EPO-producing cells and hypoxia induction of EPO production results mainly from the increase in the number of cells producing EPO (Tan et al., 1991; Eckardt et al., 1993; Juul et al., 1998; Obara et al., 2008). EPO expression is detected in tissues beyond liver and kidney including brain and neural cells, spleen, lung, and bone marrow (Fandrey and Bunn, 1993; Masuda et al., 1994; Marti et al., 1996; Dame et al., 1998; Juul et al., 1998), but does not substitute for the required erythropoietic regulation provided by the kidney. Interestingly, genetically over-stabilizing the hypoxic response in osteoblasts in mice resulted in selective expansion of the erythroid lineage leading to development of severe polycythemia due to high level of EPO expression in osteoblasts compared to relatively lower levels of EPO induced by hypoxia in control animals (Rankin et al., 2012).

### EPO Is Hypoxia Inducible

Erythropoietin production is hypoxia responsive mediated via binding of HIF to the hypoxic responsive element located downstream of the coding region under hypoxic conditions (Semenza, 2009). HIF is a heterodimer between HIF-α (HIF-1α, HIF-2α or HIF-3α) and HIF-1β (or ARNT), and HIF-2α (or EPAS1) is particularly associated with EPO regulation (Rankin et al., 2007; Suzuki et al., 2017). Oxygen dependent hydroxylases, PHD and FIH-1 down regulate HIF-α stability/activity and provide oxygen sensitivity for HIF regulation of EPO expression (Jaakkola et al., 2001; Mahon et al., 2001; Lando et al., 2002). At normoxia, HIF-α is marked for degradation by proline hydroxylation, primarily by PHD2 (Jaakkola et al., 2001), providing a binding site for VHL which targets HIF-α for ubiquitination and proteasome degradation (Ohh et al., 2000; Ang et al., 2002; Minamishima et al., 2008; Takeda et al., 2008; Kobayashi et al., 2016). With reduced oxygen, HIF-α is stabilized and increased HIF-2α in renal EPO-producing cells up regulates EPO gene expression (Semenza, 2009). The asparaginyl hydroxylase, FIH-1 binds the HIF-α transactivation domain at normoxia and inhibits HIF-α transactivation by hydroxylating asparagine residue in the carboxy-terminal transactivation domain and blocks interactions with coactivator proteins (Mahon et al., 2001; Lando et al., 2002). Mutations in VHL, PHD2, and HIF-2α have been identified in patients with familial erythrocytosis. The Chuvash population of the Russian Federation is associated with a high prevalence of polycythemia due to VHL gene mutation that reduces oxygen dependent HIF-2α degradation and increases EPO production (Ang et al., 2002; Pastore et al., 2003). Mutations in HIF-2α or the EGLN1 gene that encodes PHD2 also give rise to erythrocytosis associated with increased EPO production (Percy et al., 2006, 2008).

### EPOR Gene Regulation in the Erythroid Lineage

**FIGURE 2:**
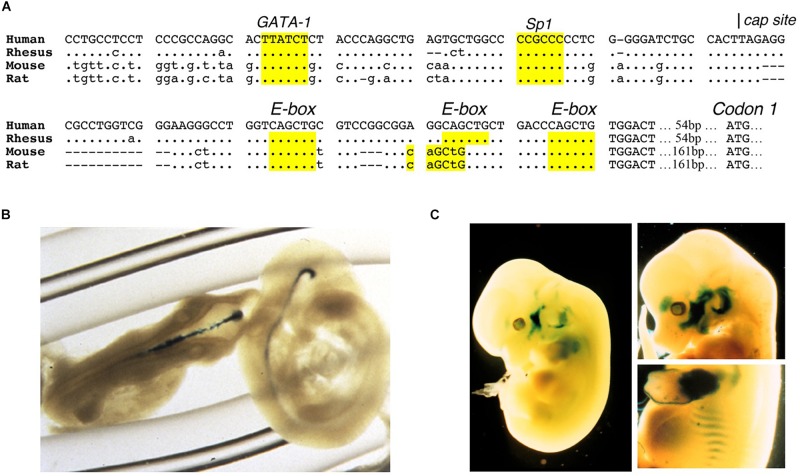
Expression of EPOR reporter gene in transgenic mice. **(A)** The proximal promoter region of the human EPOR gene extending to the translation start site with ATG at +135 in the human EPOR gene, contains conserved regulatory binding sites for GATA proteins (AGATAA) and Sp1 (CCGCCC), and 3-E-boxes (CAGCTG) in the 5′ untranslated transcribed region that can bind basic-helix-loop-helix transcription factors such as erythroid TAL1 and skeletal muscle transcription factors Myf5 and MyoD. **(B)** Transgenic mice containing the human EPOR proximal promoter region extending 1778 bp 5′ of the transcription start site driving the β-galactosidase reporter gene shows EPOR expression in the embryonic brain at embryonic day E9.5 (from Liu et al., 1997, with permission). **(C)** Reporter gene expression at embryonic day 12.5 (left) and embryonic day 13.5 (right) in the visceral arches, base of limbs, intercostal rib regions, and fetal liver (from Ogilvie et al., 2000, with permission).

Erythropoietin receptor is expressed at the highest level on erythroid progenitor cells at the colony forming unit-erythroid (CFU-E) stage that becomes the most responsive to changes in EPO level (Broudy et al., 1991). EPO is required for erythroid progenitor cell survival as cells differentiate from early erythroid progenitors or burst forming unit-erythroid (BFU-E) to CFU-E. Mice that lack EPO or its receptor die *in utero* at day 13.5 due to severe anemia (Wu et al., 1995; Lin et al., 1996). EPO binding to its receptor on erythroid progenitor cells increases expression of erythroid transcription factors, GATA1 and the basic-helix-loop-helix protein, TAL1, that in turn transactivate EPOR expression; hence, EPO regulates expression of its own receptor (Zon et al., 1991; Kassouf et al., 2010; Rogers et al., 2012). The EPOR promoter region contains conserved binding sites for ubiquitous Sp1 transcription factor and GATA1 (AGATAA) and in the 5′ untranslated transcribed region 3 E-box (CAGCTG) TAL1 binding sites (Figure 2A). EPO binding at the early erythroid progenitor BFU-E stage with low level EPOR induces GATA1 and TAL1 to activate the erythroid program including EPOR. EPOR is down regulated with progression of erythroid differentiation and is not detected on reticulocytes (Broudy et al., 1991).

### EPOR Structure and Signaling

Erythropoietin receptor is a member of the class I cytokine receptor superfamily that contains a WSXWS motif in the extracellular domain, a single transmembrane domain, a cytoplasmic domain that lacks tyrosine kinase activity and associates with JAK kinase, and form complexes that are homodimeric, heterodimeric or heterotrimeric (Bazan, 1990; Liongue et al., 2016). EPO binding to its homodimeric receptor complex brings the cytoplasmic associated JAK2 kinases in close proximity allowing for JAK transphosphorylation, phosphorylation of the receptor, and phosphorylation and activation of STAT and other downstream signaling pathways including AKT and ERK1/2 (Witthuhn et al., 1993; Miura et al., 1994; Watowich, 2011; Kuhrt and Wojchowski, 2015). Phosphorylated STAT5 subunits, STAT5A, and STAT5B, dimerize and translocate to the nucleus to activate select gene expression (Socolovsky et al., 2001).

## EPO Response Beyond Erythroid Cells

Availability of recombinant human EPO facilitated discovery of EPO response in non-hematopoietic tissue. These include endothelial cells and the cardiovascular system, neural cells and the brain, myoblasts and skeletal muscle, adipocytes and fat depots, and bone (; McGee et al., 2012; Zhang et al., 2014; Hiram-Bab et al., 2017; Figure 1).

### Endothelial EPO Response

Erythropoietin activity beyond erythropoiesis was first observed in endothelial cells. Cultures of primary endothelial cells from human umbilical vein and bovine adrenal capillary exhibited EPO binding and dose dependent proliferative and chemoattractant response to EPO, and EPOR is expressed in endothelial cells (Anagnostou et al., 1990, 1994). Prior to death *in utero*, mice that lack EPO or EPOR exhibit angiogenic defects including decreased vessel networks (Kertesz et al., 2004).

In endothelial cells, endothelial nitric oxide (NO) synthase (eNOS or NOS3) produces NO to regulate vascular tone and blood pressure. EPO stimulation of endothelial cells activates eNOS and NO production, particularly at reduced oxygen (Beleslin-Cokic et al., 2004, 2011). Transgenic mice expressing high level of human EPO with hematocrit of about 80% also exhibit markedly increased eNOS level and NO production (Ruschitzka et al., 2000). EPO induction by hypoxia or ischemic stress stimulates production of red blood cells to improve oxygen delivery and increase transport of oxygen from the lungs to the tissues. However, increased red blood cell production requires increased proliferation and differentiation of erythroid progenitor cells in bone marrow (and spleen in mice) to expand the erythroid lineage. EPO stimulated induction of NO provides the potential for an acute response regulating vascular tone and improving oxygen delivery.

Erythropoietin stimulation of endothelial cells increases ET-1 secretion that may exacerbate adverse vascular effects (Carlini et al., 1993; Barhoumi et al., 2014). Mice expressing high level of transgenic human EPO with elevated hematocrit and increased NO production also have elevated ET-1 levels, but do not exhibit hypertension (Ruschitzka et al., 2000). Exposure to NO synthase inhibitor (N-nitro-L-arginine methyl ester) decreases eNOS production of NO, increases vasoconstriction and hypertension, and decreases survival of these mice, while pretreatment with ET(A) receptor antagonist (darusentan) improves survival, indicating that increased NO production in these mice suppresses the adverse effects associated with increased ET-1 (Quaschning et al., 2003).

### EPO and Cardioprotection

During embryonic development, mice that lack EPO signaling exhibit ventricular hyperplasia indicating defect in proliferation and expansion of the myocardium (Wu et al., 1999; Yu et al., 2001). In studies of isolated adult hearts from rodents, EPO promoted cardioprotection in ischemia reperfusion injury (Cai et al., 2003; Parsa et al., 2003) and EPO administration in rabbits was cardioprotective from myocardial infarction (Cai and Semenza, 2004). In adult rats, EPO administration immediately after myocardial infarction reduced infarct size and improved cardiac function linked to neovascularization (Van Der Meer et al., 2005). Furthermore, EPO treatment in a rat model for chronic heart failure suggested that long term EPO treatment stimulated homing of endothelial progenitor cells to induce neovascularization (Westenbrink et al., 2006). Delayed weekly EPO treatment beginning 7 days after coronary occlusion in rats resulted in reduced injury and improved cardiac function associated with mobilized endothelial progenitor cells (Prunier et al., 2007). Cardioprotective effects of EPO is mediated via induction of coronary endothelial production of NO and require activation of eNOS (Mihov et al., 2009). In genetically modified mice with EPOR restricted to hematopoietic and endothelial cells, EPO cardioprotection was observed in acute ischemia reperfusion injury in heart (Teng et al., 2011a). Cardioprotection was comparable to that observed in wild type mice, but not in eNOS knockout mice, providing evidence that EPOR expression in endothelial cells was sufficient for EPO protection in ischemia reperfusion injury in heart and that activation of eNOS and increased NO production is required (Teng et al., 2011a). In addition, high hematocrit associated with chronic EPO treatment could offset the EPO cardioprotective effect.

### EPO and Neuroprotection

Erythropoietin receptor expression in neurons and select locations in brain (Masuda et al., 1993; Digicaylioglu et al., 1995; Morishita et al., 1997) combined with EPO production in astrocytes and neurons in a hypoxia inducible manner (Masuda et al., 1994; Marti et al., 1996) provide evidence for EPO signaling on the other side of the blood brain barrier. During mouse development, the brain expresses high level of EPOR mid-gestation, localized using reporter gene expression, and mice that lack EPOR exhibit thinning of the neuroepithelium, reduced neural progenitor cells with increased sensitivity to hypoxia, and increased brain apoptosis prior to embryonic death due to severe anemia (Liu et al., 1997; Yu et al., 2002; Figure 2B). Mice with EPOR expression restricted to hematopoietic and endothelial tissue and mice with targeted deletion of EPOR in neural cells show no gross morphological defects but do exhibit reduced neural cell proliferation and viability, and increased susceptibility to glutamate damage and stroke (Tsai et al., 2006; Chen et al., 2007). Conversely, EPO infusion into adult rodent brain increases the number of newly generated interneurons (Shingo et al., 2001). In animal models of brain injury, preconditioning with EPO infusion is protective for ischemia or middle cerebral artery occlusion – induced learning disability and neuron death (Sadamoto et al., 1998; Sakanaka et al., 1998; Bernaudin et al., 1999). In a rodent model of neonatal hypoxia/ischemia, EPO protection was associated with brain revascularization and neurogenesis (Iwai et al., 2007).

For clinical application of EPO for ischemic injury, a Phase I clinical trial for EPO treatment of ischemic stroke provided evidence for the safety and potential efficacy showing an association with improvement in clinical outcome at 1 month (Ehrenreich et al., 2002). A following Phase II/III clinical trial of EPO treatment with acute ischemic stroke treated 460 patients with EPO or placebo. Unexpectedly, 63% received recombinant tissue plasminogen activator for treating systemic thrombolysis that was not approved for use in ischemic stroke at the time of the Phase I trial. Favorable effects of EPO were not demonstrated, and overall death rate was 1.8 times higher in the EPO group than in the placebo group (Ehrenreich et al., 2009). Further subgroup analysis including post stroke biomarkers suggested that patients with ischemic stroke not receiving thrombolysis likely benefited from EPO treatment (Ehrenreich et al., 2009, 2011). This negative German Multicenter EPO Stroke Trial underscores the complexity posed by standard of care and difficulties in translating positive results from animal studies to the clinic.

### Alternate EPO Receptors and EPO Derivatives

High hematocrit resulting from chronically administered EPO, particularly at high dose, is associated with adverse effects such as hypertension and thromboembolism and could counteract the neuroprotective and cardioprotective effects of EPO. The potential to activate EPO/EPOR protective response in non-hematopoietic tissue via an alternate EPO receptor or an EPO mimetic without increasing erythropoietic activity and hematocrit is of particular interest. An alternate EPO receptor has been proposed for non-hematopoietic tissues such as brain and heart, consisting of a heterodimer between EPOR and the beta common (βc) receptor, also a member of the class I cytokine receptor superfamily and shared by receptors for granulocyte-macrophage colony stimulating factor and interleukins 3 and 5 (Jubinsky et al., 1997; Brines et al., 2004). EPO protection in a model of experimental colitis in mice is proposed to be mediated via activation of the EPOR/βc receptor heterodimer (Nairz et al., 2017). The role of the βc receptor in EPO response is controversial and evidence of direct interaction between EPOR and the βc receptor is lacking. In EPO responsive neural SH-Sy5y and PC12 cells, βc receptor is below the level of detection, and in rat brain, βc receptor does not colocalize with either EPO or EPOR (Nadam et al., 2007; Um et al., 2007). Furthermore, cardioprotection in mice by darbepoetin, a long acting derivative of EPO, did not require the βc receptor (Kanellakis et al., 2010). Biophysical analyses show that the extracellular domains of EPOR and the βc receptor do not directly interact in the presence or absence of EPO (Cheung Tung Shing et al., 2018), and the role of the βc receptor in EPOR response to EPO remains uncertain. Other proposed EPO binding receptors include the Ephrin B4 receptor on cancer cells and the orphan cytokine receptor CRLF3 on insect neural cells (Pradeep et al., 2015; Hahn et al., 2017).

Erythropoietin derivatives that can promote non-hematopoietic tissue protective effects, especially neuroprotection and cardioprotection, without stimulating erythropoiesis, have been proposed to bind alternate EPO receptors such as the EPOR/βc receptor heterodimer. These include asialoerythropoietin, carbamylated EPO, ARA 290 [cibinetide; helix B surface peptide (11 amino acid peptide derived from the EPO sequence)], and recombinant EV-3 (an EPO derived a spliced variant with exon 3 deleted) (Erbayraktar et al., 2003; Brines et al., 2004, 2008; Fiordaliso et al., 2005; Robertson et al., 2013; Bonnas et al., 2017). Clinical studies explored the safety and use of EPO and carbamylated EPO to increase frataxin levels for treatment of Friedreich’s Ataxia (Boesch et al., 2014; Egger et al., 2014; Santner et al., 2014). ARA 290 treatment in phase 2 trials for Sarcoidosis-associated small nerve fiber loss showed improved abundance of corneal nerve fiber and neuropathic pain following 28 day treatment (Dahan et al., 2013; Culver et al., 2017). Another phase 2 trial focused on the potential benefit of ARA 290 treatment in subjects with type 2 diabetes for neuropathy as well as metabolic control (Brines et al., 2015). These EPO derivatives suggest the potential to therapeutically activate the tissue-protective activity associated with EPO while minimizing the risk attributed to increases in erythropoiesis and hematocrit.

### Skeletal Muscle EPO Response

Reporter gene expression in transgenic mice during development revealed EPOR expression shared resemblance with the expression pattern in developing muscle associated with E-box binding basic-helix-loop-helix muscle transcription factors, MyoD and Myf-5, localizing in the visceral arches, proximal forelimb and intercostal area (Sadamoto et al., 1998; Figure 2C). Primary satellite cells isolated from mouse and human skeletal muscle express EPOR (Ogilvie et al., 2000; Rundqvist et al., 2009). Like erythroid progenitor cells, EPO increases proliferation of C2C12 myoblasts and induces EPOR expression that decreases with cell differentiation (Ogilvie et al., 2000). EPOR expression is regulated by GATA3, GATA4, and TAL1, and can also be transactivated by MyoD and Myf-5 (Ogilvie et al., 2000; Wang et al., 2012). EPO stimulates myoblast proliferation and survival, increases GATA4 and TAL1 that retard myogenic differentiation, mediated in part by Sirt1 activity, and inhibits expression of myogenin associated with differentiating myoblasts and myotube formation (Wang et al., 2012). Improved survival was demonstrated by transplantation of myoblasts over-expressing EPOR into skeletal muscle (Jia et al., 2009). In skeletal muscle, Pax-7^+^ satellite cells can self-renew or differentiate to Myf5^+^ committed muscle progenitor cells that contribute to growth, maintenance and repair of skeletal muscle (Kuang et al., 2007). Mice that express high transgenic human EPO have increased skeletal muscle Pax-7^+^ satellite cells and isolated primary myoblasts had enhanced proliferation in culture compared to wild type cultures (Jia et al., 2012). These mice subjected to skeletal muscle injury exhibited improved muscle repair and recovery and increased maximum load tolerated by isolated muscle. In contrast, ΔEpoR_E_ have fewer Pax-7^+^ satellite cells and isolated primary myoblasts from ΔEpoR_E_ mice do not proliferate in culture, and in skeletal muscle injury, these mice show delayed muscle repair and recovery, and reduced maximum load tolerated by isolated muscle (Jia et al., 2012). Furthermore, EPO treatment increases Pax-7^+^satellite cells and promotes repair and recovery from skeletal muscle injury (Jia et al., 2012). Skeletal muscle myoblasts produced endogenous EPO that increased at low oxygen, and transgenic mice with high transgenic human EPO exhibit mouse EPO and elevated human EPO expression in primary myoblasts, raising the possibility of an autocrine EPO response in skeletal muscle (Jia et al., 2012). Transgenic knockdown of circulating EPO levels did not show any change in EPOR gene expression in mouse skeletal muscle (Hagström et al., 2010; Mille-Hamard et al., 2012).

In humans, a single injection of EPO increased myogenic regulatory factor *MYF6* mRNA (Lundby et al., 2008) and EPO treatment increased PAX7 and MYOD1 content in human satellite cells (Hoedt et al., 2016), suggesting a role for EPO and its receptor in muscle development or remodeling. EPO administration also enhanced muscle mitochondrial oxidative phosphorylation and electron chain transport capacity following 8 weeks of treatment (Plenge et al., 2012). EPO stimulation in C2C12 myoblasts increased JAK2, STAT5 (Sadamoto et al., 1998) and AKT phosphorylation (Jia et al., 2009), and mice expressing elevated EPO in skeletal muscle by gene transfer also exhibited increased AKT phosphorylation (Hojman et al., 2009). In humans, a single EPO injection followed by exercise was not sufficient to activate the AKT pathway in skeletal muscle (Lamon et al., 2016).

### EPO and Skeletal Muscle Fiber Type

Skeletal muscles of vertebrates contain mainly two types of muscle myofibers, type I (slow twitch) and type II (fast twitch) that differ in their function, mitochondrial density, and metabolic properties (Zierath and Hawley, 2004). Type I muscle fibers contain a high concentration of mitochondria and high oxidative capacity, and exhibit fatigue resistance and prolonged duration of muscle activity (Zierath and Hawley, 2004). Endogenous EPO contributes to muscle myofiber type. In ΔEpoRE mice with EPO activity restricted to erythroid tissue, skeletal muscles exhibit fewer type I muscle fibers and reduced mitochondrial activity (Wang et al., 2013a). In contrast, skeletal muscles from transgenic mice with high EPO production with high transgenic EPO production show an increase in the proportion of type I muscle fibers and increased mitochondrial activity (Wang et al., 2013a). Furthermore, mice with high EPO production show prolonged position holding time to an inverted wire grid, suggesting that elevated EPO results in improved muscle response to fatigue (Jia et al., 2012). PGC-1α is expressed mainly and preferentially in type I muscle fibers and activates mitochondrial biogenesis and oxidative metabolism in mice (Lin et al., 2002). EPO treatment of primary skeletal myoblast cultures increases mitochondrial biogenesis gene expression including PGC-1α, increased cytochrome C and oxygen consumption rate that can contribute to skeletal muscle fiber programming and development of type I muscle fibers (Wang et al., 2013a).

## EPO Protection in Diet Induced Obesity

Erythropoietin regulation of metabolism extends beyond oxygen delivery and contributes to maintenance of white adipose tissue and metabolic homeostasis. Diet-induced obesity gives rise to glucose intolerance and insulin resistance, leading to type 2 diabetes. Animal studies suggest that EPO may be protective in diet-induced obesity, improves glucose tolerance, reduces insulin resistance and regulates fat mass accumulation, particularly in male mice (Wang et al., 2014; Zhang et al., 2014; Alnaeeli and Noguchi, 2015).

### EPO and Inflammation in Obese White Adipose Tissue

The anti-apoptotic and protective effects of EPO in select tissues such as adult and preterm brain contribute to an anti-inflammatory response, inhibiting expression of proinflammatory cytokines and reducing macrophage infiltration (Villa et al., 2003; Wassink et al., 2017). The immune-modulatory activity of EPO as observed in the gut is mediated by JAK2 activation and inhibition of macrophage NF-kB response (Nairz et al., 2011). In diet-induced obesity, EPO modulates the proinflammatory response of macrophage infiltration in white adipose tissue and promotes an anti-inflammatory phenotype (Alnaeeli et al., 2014).

In white adipose tissue, macrophages are found in the stromal vascular fraction. High fat diet feeding in C57BL/6 mice results in inflammation and macrophage infiltration of white adipose tissue, resulting in crown-like structures of macrophages surrounding necrotic adipocytes. Among non-hematopoietic tissues, EPOR is highly expressed in white adipose tissue and specifically on adipocytes and macrophages in the stromal vascular fraction, and EPO treatment exhibits anti-inflammatory activity in white adipose tissue of obese mice (Alnaeeli and Noguchi, 2015). In obese C57BL/6 male mice, short term EPO treatment (2 weeks) increases hematocrit without affecting body mass, improves glucose tolerance and insulin resistance, and shifts the inflammation of white adipose tissue associated with high fat diet feeding toward an anti-inflammatory phenotype (Alnaeeli et al., 2014). EPO treatment reduces inflammation and crown-like structures in white adipose tissue, decreases pro-inflammatory cytokine/chemokine expression, and increases anti-inflammatory cytokine interleukin 10 expression. EPO reduces the total number of macrophages and shifts the remaining macrophage population toward the anti-inflammatory macrophage subtype. EPO promotes STAT3 phosphorylation in white adipose tissue macrophages and the EPO stimulated increase in the anti-inflammatory macrophage subtype is dependent on interleukin 4/STAT6 signaling. Furthermore, ΔEpoR_E_ lack EPOR expression in white adipose tissue and exhibit higher circulating inflammatory monocytes on high fat diet feeding compared with wild type mice, suggesting a role for immune regulation by endogenous EPO/EPOR signaling (Alnaeeli and Noguchi, 2015). ΔEpoR_E_ mice on high fat diet show even greater inflammation and crown-like structures in white adipose tissue, elevated cytokine/chemokine expression in perigonadal white adipose tissue stromal vascular fraction, glucose intolerance and insulin resistance. When treated with EPO, ΔEpoR_E_ mice exhibit the expected increase in hematocrit without significant difference in glucose tolerance or inflammation in white adipose tissue (Alnaeeli et al., 2014).

Insulin resistance associated with diet induced obesity has been linked to inflammation of white adipose tissue (Lumeng and Saltiel, 2011; Han and Levings, 2013; Chen et al., 2019). This suggests that the activity of EPO to reduce white adipose tissue inflammation in diet induced obesity may contribute to EPO stimulated improvement in insulin resistance. Other EPO associated metabolic activity can also affect insulin resistance. Adipocyte response to EPO contributes to insulin sensitivity and C57BL/6 mice with adipocyte-specific deletion of EPOR on high fat diet exhibit decreased glucose tolerance and insulin sensitivity, an effect that may depend on mouse background strain (Luk et al., 2013; Wang et al., 2013b). In pancreatic β-cells EPO exerts JAK2 dependent protective effects and induces proliferative, anti-inflammatory and angiogenic activity within the islets in mouse diabetic models (Choi et al., 2010). EPO enhances AKT activation in liver, inhibits gluconeogenesis in high fat diet fed mice and reduces liver inflammation associated with diet induced obesity (Meng et al., 2013). EPO activity in brain, particularly the hypothalamus, also influences metabolic homeostasis (Teng et al., 2011b; Dey et al., 2016).

### Endogenous EPO Is Required for Erythropoiesis and Regulates Fat Mass Accumulation

Expression of EPOR beyond erythroid tissue and the protective effects of EPO administration in animal models of ischemia and traumatic injury in non-hematopoietic tissue such as the cardiovascular system, brain and skeletal muscle raise questions about the requisite role of EPO beyond regulation of red blood cell production. ΔEpoR_E_ mice with EPOR expression restricted to erythroid tissue were created using an erythroid specific transgene expressing EPOR cDNA driven by the erythroid transcription regulatory regions of the GATA1 erythroid transcription factor to rescue the EPOR knockout mouse (mEPOR-/-) (Suzuki et al., 2002). These mice survive through adulthood, providing evidence that the primary and necessary function of EPO is to regulate red blood cell production, and that the non-hematopoietic EPO activity is dispensable for embryonic development.

**FIGURE 3:**
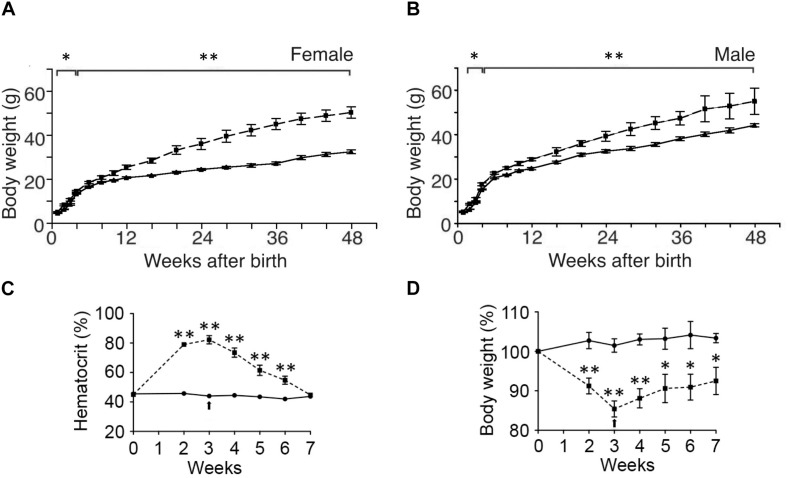
Endogenous and exogenous EPO signaling regulates white fat mass accumulation. **(A,B)** Body weight to 48 weeks of age are indicated for wild type mice (solid line) and mice with EPOR restricted to erythroid tissue (Suzuki et al., 2002) (ΔEpoR_E_) (dashed line) for females **(A)** and males **(B)**. **(C,D)** Hematocrit **(C)** and % body weight normalized to starting body weight **(D)** for male wild type mice subjected to 3 weeks of EPO treatment at 3000 U/kg three times weekly (dashed line) or saline (solid line). Arrow indicates end of EPO treatment. ^∗^*p* < 0.05; ^∗∗^*p* < 0.01 (from Teng et al., 2011b, with permission).

While ΔEpoR_E_ mice created on a C56BL/6 background exhibit no gross morphologic defects, they exhibit a disproportionate accumulation of fat mass with age (Teng et al., 2011b). By 4 months, the body mass is 60% greater in female ΔEpoR_E_ mice than wild type control mice and 25% greater in male ΔEpoR_E_ mice than wild type control mice (Figures 3A,B). The increase in body mass in ΔEpoR_E_ mice is due to an increase in both the visceral and subcutaneous white fat. Fat mass continues to disproportionately increase in ΔEpoR_E_ mice and by 8 months, fat mass is more than doubled in female ΔEpoR_E_ mice compared to control. ΔEpoR_E_ mice are glucose intolerant and with increasing accumulation of fat mass become insulin resistant by 4 months in female and by 6 months in male mice. While there is no difference in food intake by ΔEpoR_E_ mice on normal chow, ΔEpoR_E_ mice exhibited greater body weight gain normalized to food intake, consistent with decreased energy expenditure. Even before overt obesity, young ΔEpoR_E_ mice exhibited decreased locomotor activity and decreased metabolic rate assessed by indirect calorimetry. On high fat diet, ΔEpoR_E_ mice behaved similarly with food intake comparable to wild type control mice and had decreased locomotor activity and metabolic rate.

C57BL/6 mice with targeted deletion of EPOR in adipocytes fed normal chow exhibited a modest increase in body weight and fat mass with lower total activity and oxygen consumption (Wang et al., 2013b). High fat diet feeding further accentuated these differences, although food intake was comparable with wild type control mice. Young mice that lack EPOR in adipocytes fed high fat diet for 6 weeks showed a 1.4-fold increase in fat mass compared with control mice and higher blood glucose and serum insulin levels, glucose intolerance and insulin resistance, suggesting that endogenous EPOR expression on adipocytes contributes significantly to metabolic regulation.

### EPO Activity in Adipocytes and Regulation of Fat Mass Accumulation

Initial studies in mice suggesting EPO activity in metabolic homeostasis involved skeletal muscle gene transfer to over-express EPO that resulted in weight reduction in obese mice due to reduction in fat mass accompanied by increased muscle oxidation and normalization of glucose sensitivity (Hojman et al., 2009; Teng et al., 2011b). Further studies of exogenous EPO treatment in mice including genetic mouse models of obesity and transgenic mice constitutively overexpressing human EPO showed that elevated serum EPO levels decreased blood glucose and decreased body weight, especially body weight gain in obese mice (Katz et al., 2010). Interestingly, hemodialysis patients on short-term EPO treatment showed improved glucose metabolism indicated by a reduction in insulin resistance and decreased glycated hemoglobin and hyperleptinemia (Osman et al., 2017). Accompanying the increase in hematocrit with EPO treatment in C57BL/6 male mice is a decrease of body weight in mice fed normal chow and a reduction in weight gain and fat mass accumulation in mice on high fat diet feeding (Teng et al., 2011b; Figures 3C,D). However, ΔEpoR_E_ treated with EPO show the expected increase in hematocrit without change in body weight indicating that EPO regulation of fat mass is independent of EPO stimulated red blood cell production (Teng et al., 2011b). Mice with selective deletion of EPOR on adipocytes exhibit increased hematocrit with EPO treatment, but only a non-significant decreasing trend in body weight, exemplifying the direct role of EPO response in adipocytes to EPO regulation of body weight and fat mass accumulation in addition to glucose metabolism and insulin sensitivity (Wang et al., 2013b).

In white adipose tissue, EPOR is expressed at high level (about 60% of spleen, a mouse hematopoietic tissue), and in culture, EPO treatment decreases preadipocyte differentiation and induces ERK activation in primary mouse embryonic fibroblasts but not in embryonic fibroblasts generated from ΔEpoR_E_ mice (Teng et al., 2011b). Despite the increase in fat mass in ΔEpoR_E_ mice, analysis of adipocyte size distribution in gonadal fat pads showed a shift to smaller cells in ΔEpoR_E_ mice indicating a marked increase in adipocyte number with loss of EPOR in non-hematopoietic tissue, providing further evidence that endogenous EPO contributes to regulation of adipocyte number in addition to fat mass accumulation (Teng et al., 2011b).

Insulin stimulates AKT activation and analysis of white adipose tissue from male C57BL/6 mice revealed that EPO treatment also increased AKT phosphorylation in white adipose tissue, but not in mice with targeted deletion of EPOR in adipocytes, suggesting that EPO modulates AKT activation in white adipose tissue and potentially affects insulin signaling (Wang et al., 2013b). Further analysis of white adipose tissue shows that EPO treatment promotes a brown fat-like program, increases mitochondrial biogenesis independent of changes in body weight, and increases cellular respiration rate, and decreases the white fat-like program (Wang et al., 2013b). Conversely, white adipose tissue from mice with targeted deletion of EPOR in adipocytes shows decreased mitochondrial biogenesis, decreased cellular respiration rate and suppression of brown fat-like program and increase in white fat-like program, and no response to EPO stimulation (Wang et al., 2013b). EPO activity in white adipocytes is mediated via increased PPARa that cooperates with increased activity of metabolic sensor Sirt1, an NAD-dependent class III histone deacetylase sirtuin.

In addition to EPO regulation of fat mass accumulation in white adipose tissue, EPO decreased lipid accumulation in the liver while stimulating STAT3/STAT5 activation and promoting lipolysis in white adipose tissue, suggesting benefit in non-alcoholic fatty liver disease (Tsuma et al., 2019). In brown adipose tissue of young C57BL/6 mice, EPO treatment stimulated STAT3 and upregulated transcription factor PRDM16 that controls brown adipocyte differentiation and total UCP1 that is essential for brown adipose tissue thermogenesis (Kodo et al., 2017).

### EPO Regulation of Proopiomelanocortin (POMC) and Food Intake

Erythropoietin treatment in male mice fed high fat diet showed both increase in activity and decrease in food intake, pointing to potential EPO regulation in the central nervous system to regulate food intake and energy expenditure (Teng et al., 2011b). The hypothalamus regulates appetite by production in the arcuate nucleus of the orexigenic or appetite-increasing neuropeptide Y and agouti-related protein, and the anorexigenic or appetite-suppressing precursor protein, POMC. EPOR is expressed in brain, and EPOR expression level in hypothalamus is comparable to white adipose tissue and EPOR in the hypothalamus colocalizes to POMC expressing neurons in the arcuate nucleus (Teng et al., 2011b). EPO treatment in C57BL6 male mice increases hypothalamus expression of POMC and α-MSH, a POMC cleavage product, but not expression of neuropeptide Y or agouti-related protein (Teng et al., 2011b; Dey et al., 2016). Leptin stimulates STAT3 activation in the hypothalamus resulting in production of several neuropeptides including POMC to regulate appetite; hypothalamus neural cultures show that EPO also activates STAT3 (Dey et al., 2016). Conversely, ΔEpoR_E_ mice show a decrease in STAT3 activation in hypothalamus and reduced POMC levels with and without EPO treatment, suggesting that EPO regulates appetite, and potentiates leptin response (Dey et al., 2016).

In the pituitary, POMC production gives rise to ACTH. Although EPO treatment in wild type mice increases hypothalamus production of POMC, EPO treatment decreases plasma ACTH level (Dey et al., 2015). On the other hand, plasma concentration of ACTH is high in ΔEpoR_E_ mice with reduced POMC production in the hypothalamus, suggesting that both endogenous and exogenous EPO contributes to regulation of plasma ACTH. EPO treatment in cultures of mouse corticotroph pituitary cell line shows decrease in basal intracellular calcium levels, no change in POMC mRNA and increased intracellular ACTH, indicating disruption of post-translational processing of POMC and inhibition of ACTH secretion. EPO regulation of pituitary derived ACTH plasma levels suggests a wider role for EPO regulation of metabolism and obesity via the neuroendocrine hypothalamic-pituitary axis (Dey and Noguchi, 2017).

### Gender Specificity of EPO Action and Regulation of Fat Mass

Estrogen can affect EPO action via direct regulation of EPO production or modulation of tissue specific EPO response. For example, in adult female mice, estrogen dependent induction of EPO in the mouse uterus contributes to angiogenic activity and blood vessel formation in the uterine endometrium, contributing to the cyclic remodeling in the estrus cycle transition from diestrus to proestrus (Yasuda et al., 1998). With regards to environmental hypoxia, EPO affects the hypoxic ventilatory response via EPOR expression in brain and carotid body, and this response is increased in women and female mice compared with men and male mice (Soliz et al., 2012). This sexual dimorphism of EPO stimulated hypoxic ventilatory response is attributed, in part, to carotid body sensitivity to sex hormones, particularly estrogen.

**FIGURE 4:**
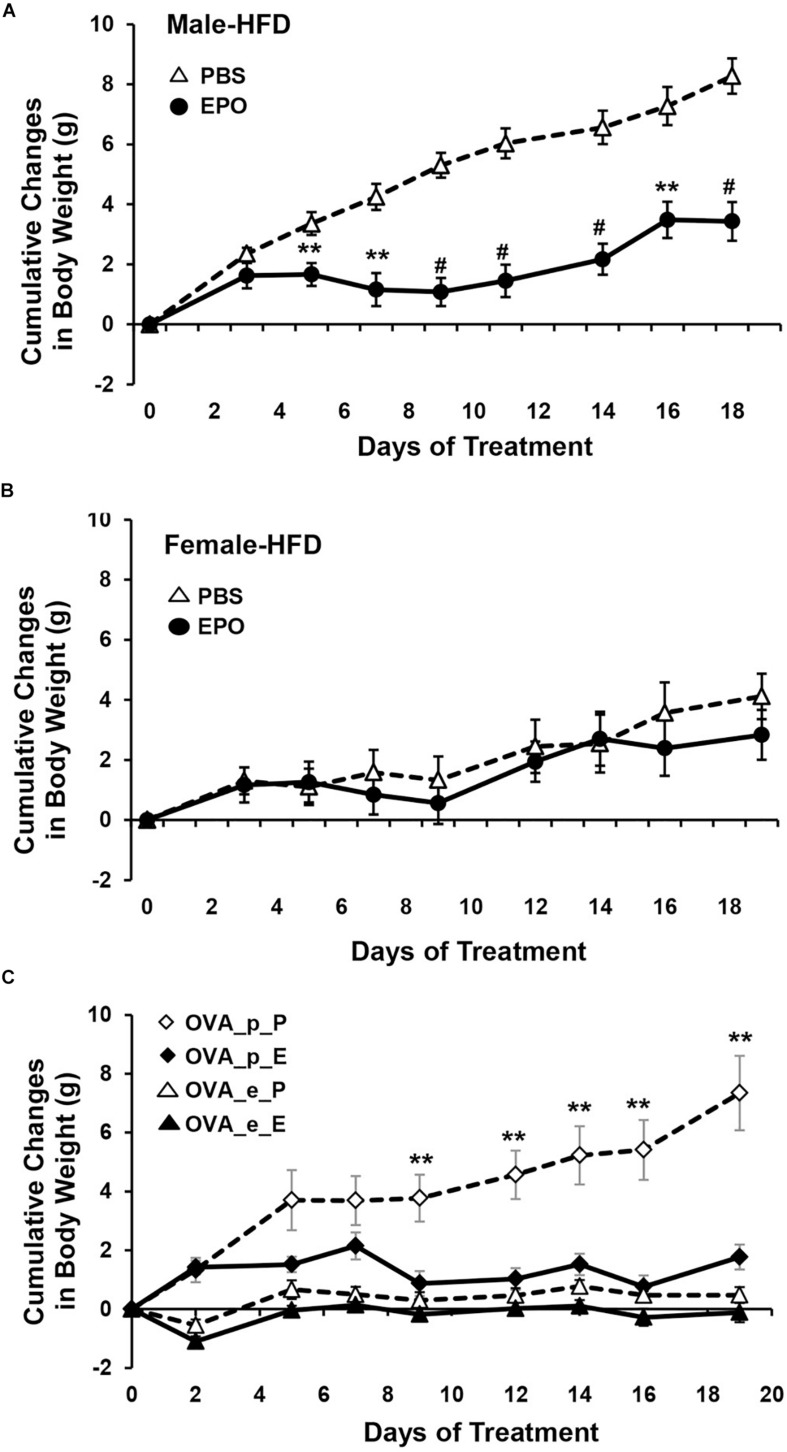
EPO regulation of fat mass is gender specific. **(A,B)** Cumulative body weight change was monitored in male **(A)** and female **(B)** mice (16 weeks) fed high fat diet and treated with EPO at 3000 U/kg three times weekly (solid symbol) or saline (open symbol) for 3 weeks. **(C)** Cumulative body weight change in female ovariectomized (OVA) mice with placebo (p) or estradiol pellet-supplement and treated with EPO (E) or phosphate-buffered saline (P). ^∗∗^*p* < 0.01; ^#^*p* < 0.001 (from Zhang et al., 2017, with permission).

Erythropoietin regulation of metabolism also appears to be sex-dependent. In ΔEpoR_E_ mice the age dependent accumulation of excess body fat is greater in female that develop obesity and insulin resistance by 4 months of age compared with male mice that exhibit a slower rate of body fat accumulation, becoming obese and insulin resistance at 6 months of age (Teng et al., 2011b; Figures 3A,B). In contrast, fat mass is not altered by EPO treatment in female C57BL/6 mice on normal chow or high fat diet, while EPO treatment in male mice on normal chow decreases fat mass and EPO treatment in male mice on high fat diet reduces the accumulation of fat mass (Zhang et al., 2017; Figures 4A,B). Gender-specific response was also observed in gene expression in white adipose tissue where EPO treatment in mice increased expression in select oxidative genes in male mice on normal chow and on high fat diet, but not in female mice. The greater increase in fat mass in male mice on high fat diet compared with female mice is evidence of the protective effect of female hormones against diet induced obesity (Figures 4A,B) and raises the possibility that female hormones interfere with the anti-obesity effect of EPO observed in male mice. The anti-obesity effect of EPO on mice fed high fat diet was restored in ovariectomized mice. Like male mice, ovariectomized mice on high fat diet treated with EPO showed the decrease in fat mass (Figure 4C). The protective effect of estrogen to diet induced obesity was demonstrated in ovariectomized mice supplemented with estradiol which was more effective in reducing fat mass than EPO, and fat mass was not further enhanced with estrogen combined with EPO (Figure 4C). EPO stimulated increase in hematocrit was comparable in male in female mice indicating that the sex-dependent regulation of fat mass is independent of EPO regulated response in erythroid tissue.

**FIGURE 5:**
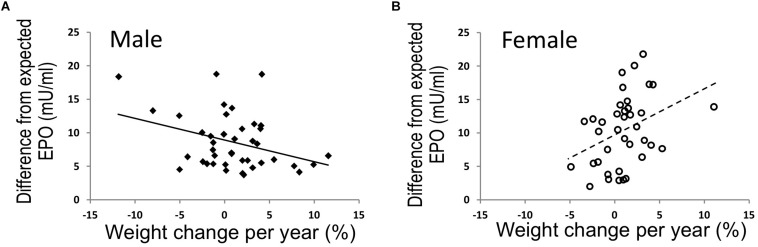
EPO and % weight change per year were associated in opposing directions in males and females. **(A,B)** In full-heritage Southwestern Native Americans, the association of plasma EPO concentrations (adjusted for creatinine, hemoglobin, and storage time) and % weight change per year was negative in men **(A)** (closed squares, *N* = 41; *r* = –0.35, *p* = 0.02). Plasma EPO concentration was positively associated with % weight change per year in females **(B)** (open circles, *N* = 38; *r* = 0.37, *p* = 0.02) (modified from Reinhardt et al., 2016, with permission).

To further examine the relationship of EPO level with body weight in human, endogenous plasma EPO concentration was assessed in a subset of full-heritage Southwestern Native Americans studied to understand the high prevalence of obesity and type 2 diabetes (Smith et al., 1996; Pavkov et al., 2007). As expected, endogenous plasma EPO level negatively associated with hemoglobin (*p* = 0.005) and no association was found for EPO and percent weight change per year in the study group of 79 individuals (Reinhardt et al., 2016). However, when segregated by sex, males exhibited an association between higher EPO concentrations and higher 24-h energy expenditure and an inverse association of endogenous EPO level with percent weight change per year (*p* = 0.02) (Figure 5A). In contrast, females exhibited a positive association of EPO plasma level with weight change per year (*p* = 0.02) (Figure 5B). Hence, endogenous EPO association with weight loss in men and with weight gain in women is distinct from EPO regulation of erythropoiesis that is comparable in both men and women and provides additional evidence for non-hematopoietic and gender-specific endogenous EPO action on regulation of body weight.

### Elevated EPO in Human and Decreasing Obesity Prevalence at Increasing Altitude

In humans, as one ascends to high altitude increased EPO production via HIF regulation induces an EPO response that increases iron utilization for hemoglobin synthesis with expansion of red blood cell production with elevated blood hemoglobin and hematocrit (Gassmann and Muckenthaler, 1985; Smith et al., 2008). The relationship between elevated EPO and reduction in fat mass suggested by metabolic studies in male mice (Teng et al., 2011b; Zhang et al., 2017), raises the possibility that increased EPO levels may explain in part the lower obesity rate reported for humans associated with residence at high altitude (Voss et al., 2013, 2014). In the United States, obesity prevalence is associated inversely with elevation and urbanization after adjusting for diet, ambient temperature, physical activity, smoking and demographic factors, and among overweight service members (proportion male of 93% at low altitude and 94% at high altitude), those stationed at high altitude were associated with lower incidence of obesity (Voss et al., 2013, 2014).

Note that high EPO levels can increase the risks of adverse effects such as cardiovascular complications and thromboembolic events, seizures, pulmonary embolism and death. For individuals in residence above 2500 m above sea level, about 5–10% can develop excessive erythrocytosis and associated Chronic Mountain Sickness (Villafuerte and Corante, 2016). However, Tibetan high-altitude natives are the exception and exhibit a different adaptive response from the hypoxia induction of EPO, allowing more efficient use of oxygen in their tissues (Horscroft et al., 2017). Even without high altitude, increase risk of adverse effects are evident with high endogenous EPO. In the elderly, elevated EPO levels were associated with increased risk of death, and in renal transplant recipients higher endogenous plasma EPO was associated with cardiovascular mortality and all-cause mortality (den Elzen et al., 2010; Sinkeler et al., 2012). Elevated EPO levels may be indicative of EPO resistance resulting from underlying co-morbidities including inflammation, iron status, malnutrition and disrupted NO metabolism (Ganz and Nemeth, 2016; Yokoro et al., 2017). The increase risk of adverse effects with high exogenous EPO administration is exemplified in extreme athletes who use EPO as a performance-enhancing drug. Around the time that EPO received FDA approval in 1989 for treatment of anemia in chronic renal disease, death associated with EPO doping was suspected in eighteen young professional cyclists who died from unknown causes (Noakes, 2004). While patients with renal failure clearly benefit from EPO treatment to correct anemia (Eschbach et al., 1987; Us Renal Data System, 2007; Hasegawa et al., 2018) several clinical trials of EPO treatment to normalize hemoglobin values in kidney disease showed increase cardiovascular morbidity and mortality associated with high dose EPO treatment (Drueke et al., 2006; Singh et al., 2006). With increased concerns about safety of high dose EPO, the FDA issued a “Black Box” warning in 2007 indicating reduced EPO dose to achieve lower target hemoglobin levels. Labeling for erythropoiesis-stimulating agents was further modified by the FDA in 2011 to lower dosing recommendations. Hence, potential benefits and potential risks must be considered with EPO treatment.

## EPO Promotes Context-Dependent Bone Remodeling

In bone, functional EPO receptors are expressed by osteoblasts and osteoclasts that remodel the bone and by BMSCs that differentiate into osteoblasts, bone marrow adipocytes and chondrocytes. Studies on the effects of EPO on these specific cell types and overall bone homeostasis have utilized *in vitro* cultures (Kim et al., 2012; Rolfing et al., 2014a; Li et al., 2015), *in vivo* BMSC osteogenic assays using ectopic ossification models (Suresh et al., 2019), bone fracture healing models (Holstein et al., 2007; Mihmanli et al., 2009; Rolfing et al., 2014b; Omlor et al., 2016), transgenic mouse model with high human EPO (model for chronic EPO exposure) (Hiram-Bab et al., 2015; Suresh et al., 2019), administration of exogenous EPO in healthy mice (Shiozawa et al., 2010; Singbrant et al., 2011; Hiram-Bab et al., 2015; Suresh et al., 2019) (model for acute EPO exposure) and, recently, ΔEpoR_E_ mice that do not express EPOR in non-erythroid cells (Suresh et al., 2019) (model for endogenous EPO signaling).

### Osteoclasts and EPO Response

Several reports including conflicting results suggest EPO regulating osteoblasts, osteoclasts and BMSCs in *in vitro* culture conditions. Osteoclast differentiation assays using marrow mononuclear cells (Shiozawa et al., 2010), non-adherent bone marrow cells (Hiram-Bab et al., 2015) and RAW264.7 mouse monocyte/macrophage cell line show EPO increasing osteoclast numbers (Li et al., 2015), but not osteoclast activity (Shiozawa et al., 2010; Li et al., 2015). However, increase in osteoclast numbers was not reported in other studies using mouse bone marrow cultures (Suresh et al., 2019) and in osteoblast-osteoclast cocultures treated with EPO (Singbrant et al., 2011). In cultures of preosteoclasts, EPO activates JAK2/PI3K pathways without affecting proliferation and in osteoclasts, EPOR expression decreases with differentiation (Hiram-Bab et al., 2015). Transgenic mice with chronic exposure to high levels of human EPO had more osteoclasts lining the bone surface (Hiram-Bab et al., 2015). Osteoclasts from these mice produced EPO and corresponding BMSC derived cultures from these mice exhibited a greater number of giant multinucleated osteoclasts (Suresh et al., 2019).

### Osteoblasts and EPO Response

For osteoblast differentiation assays, studies have utilized mouse primary calvarial osteogenic cells as well as adherent BMSCs. EPO treatment of primary mouse calvarial osteogenic cells did not affect differentiation (Hiram-Bab et al., 2015; Suresh et al., 2019). However, in transgenic mice expressing high human EPO, calvarial osteoblasts produced human EPO and showed increased ALP expression and mineralization (Suresh et al., 2019). Conversely, primary calvarial osteoblasts lacking endogenous EPO signaling had reduced ALP expression and mineralization (Suresh et al., 2019). Other osteoblast assays using human and mouse BMSCs treated with EPO reported increased osteoblast differentiation with the activation of EphrinB2/EphrinB4 (Li et al., 2015), mTOR (Kim et al., 2012), JAK2/PI3K pathways (Rolfing et al., 2014a). BMSC cultures with low EPO doses less than 5 U/mL reduced osteoblast mineralization whereas high EPO doses (50 U/mL–250 U/mL) increased the BMSC proliferation and differentiation into osteoblasts (Rauner et al., 2016). EPO stimulation of HSCs to produce BMP and thereby stimulating osteoblasts are also reported (Shiozawa et al., 2010). In a recent study using mesenchymal stem cells from young and old healthy individuals and patients with MDS, EPO treatment increased mineralization only in cells from young healthy individuals and not in cells from older healthy donors and cells from MDS patients (Balaian et al., 2018), suggesting that EPO bone remodeling activity may be age dependent. EPO treatment of mesenchymal stem cells from MDS patients inhibited Wnt signaling and reactivation of Wnt signaling combined with EPO treatment promoted their differentiation to osteoblasts (Balaian et al., 2018).

### EPO and Bone Fracture Repair

A role for EPO in bone regulation was initially reported in mouse models of fracture repair, where 5000 U/kg EPO doses for 6 days stimulated early endochondral ossification and bone mineralization along with reduced EPOR in differentiating chondrocytes (Holstein et al., 2007). A follow up study showed accelerated bone healing with much reduced dose of EPO at 500 U/kg for five-week treatment where EPO promoted endosteal vascularization and reduced NF-KB expression in the fracture callus suggesting anti-inflammatory role for EPO (Garcia et al., 2011). In alveolar bone regeneration studies in rats, EPO administration into the tooth sockets, promoted new bone formation while inhibiting bone resorption (Li et al., 2015). In rabbits with mandibular distraction osteogenesis, 150 IU/kg EPO treatment for 30 days resulted in new bone formation with increased osteoblasts, blood vessels and reduced osteoclasts (Mihmanli et al., 2009). In rabbits, implantation of gelatin sponges soaked with EPO near the bone defects followed by a single high EPO dose of 4900 IU/kg accelerated bone healing and vascularization in the callus (Omlor et al., 2016). In porcine models with calvarial defects, a single EPO dose of 900 IU/ml in collagen carrier moderately increased bone healing without any increase in vasculature (Rolfing et al., 2014b). However, in a separate study in porcine models, EPO enhanced bone healing only in combination with bone marrow concentrate containing mesenchymal stem cells (Betsch et al., 2014).

Studies have also utilized the potential of EPO in recruiting BMSCs to sites of bone healing. In rats with intratibial fracture, tail vein injection of BMSCs along with intramuscular injection of EPO was shown to mobilize BMSCs to bone defects and enhanced bone regeneration while increasing bone strength (Li et al., 2019). EPO loaded scaffolds in BALB/c mice showed increased recruitment of multipotent stem cells, this study also demonstrated that in mouse calvarial defect model, implantation of scaffolds with EPO resulted in accelerated bone healing compared to BMP2 scaffolds (Nair et al., 2013). Two-week EPO treatment of murine cranial defect models with BMP2 scaffolds implanted at the defect site also accelerated healing while promoting stem cell recruitment to the scaffolds (Sun et al., 2012). In patients with tibiofibular fracture, administration of 4000 IU of EPO in the fractures site 2 weeks after surgery had 2 week faster union and fewer non-union rates compared to the placebo group (Bakhshi et al., 2013; Nemati and Fallahi, 2013), suggesting possibility of clinical EPO use in accelerating fracture healing. Thus, using multiple species and various EPO doses and mode of administration, EPO treatment has been demonstrated to exert a beneficial role in bone healing.

### Bone Loss Concomitant With EPO Stimulated Erythropoiesis in Mice

In contrast to the bone formation potential of EPO in fracture healing studies, EPO administration in healthy mice results in significant reduction of trabecular bone volume. EPO administration in 9 week old male C57BL/6 mice for 10 days at a physiological dose of 300 U/kg resulted in significant trabecular bone loss in tibia along with increase in the number of osteoclasts and osteoblasts, indicating increased bone turnover induced by EPO treatment (Singbrant et al., 2011). However, dynamic morphometry analysis revealed that EPO administration did not affect mineral apposition rate or double labeled surface, but significantly reduced single labeled surface, suggesting a reduction in osteoblast activity (Singbrant et al., 2011). Reduction in trabecular bone coupled with increased osteoclast numbers in the femurs of 12-week-old female mouse models was also observed in chronic and acute EPO exposure (Hiram-Bab et al., 2015). Since addition of EPO increased only osteoclast differentiation *in vitro* and did not affect osteoblast differentiation in cultures, the reduced bone formation *in vivo* with EPO treatment was attributed to an indirect effect of EPO (Hiram-Bab et al., 2015). However in subsequent studies, a very low dose of EPO administration in mice was shown to significantly reduce osteoblast activity without affecting bone resorption (Rauner et al., 2016). In contrast, in mice with chronically elevated high EPO, both osteoblast and osteoclast differentiation was increased suggesting increased remodeling contributing to their reduced bone mass (Suresh et al., 2019). Thus, the dose of EPO is important in determining the effect on osteoblasts and subsequently on its impact in bone health.

Studies in transgenic mice with conditional deletion of PHD2 have shown the importance of PHD2-HIF2α-EPO signaling in bone remodeling. Low bone density due to reduced osteoblast activity without affecting osteoclasts was observed in transgenic mice with conditional deletion of PHD2 in hematopoietic lineage, renal and neural cells. In these mice PHD2 deletion resulted in excessive HIF-2α induced EPO production increasing the hematocrit to 86%. Surprisingly, deletion of PHD2 in osteoblastic lineage increased bone density and reduced osteoclast numbers *in vivo* with no change in hematocrits. While deletion of PHD2 in osteoclast lineage did not result in any bone phenotype (Rauner et al., 2016).

Reduced trabecular bone was also observed in the femurs of C57BL/6 mice with 10 day EPO administration at 1200 U/kg (Suresh et al., 2019). In contrast to these studies, increased bone formation with EPO treatment was reported in the vertebrae of young and older mice receiving supraphysiologic doses of EPO up to 6000 U/kg for three times a week for 28 days (Shiozawa et al., 2010). Of note, the reported modest increase in hematocrits with such high doses of EPO treatment has not been explained (Shiozawa et al., 2010). These changes in hematocrit are in marked contrast to the expected increase in hematocrit associated with EPO dose. For example, low dose of 150 U/kg of EPO elevated hematocrits to 60% (Foskett et al., 2011) whilst higher doses of 3000 U/kg of EPO increased hematocrits to 70% in C57BL/6 mice (Zhang et al., 2017).

Osteoblasts exhibit the potential for EPO production. In genetically modified mice, targeted deletion of VHL in osteoblasts over-stabilizes the hypoxic response and gives rise to high EPO production in osteoblasts that leads to severe polycythemia (Rankin et al., 2012). Conversely, inactivation of HIF-2 in osteoblasts resulted in decreased EPO expression in bones of neonatal mice and remained at the limit of detection in adult mice. This novel finding that osteoblasts could produce EPO raises the potential for autocrine regulation of EPO response in osteoblasts. In other studies, EPO stimulated FGF23 production in HSCs was associated with an increase in serum FGF23 and reduced serum phosphate suggesting a possible mechanism of EPO induced bone reduction due to disrupted mineralization (Clinkenbeard et al., 2017).

### Endogenous EPO Contributes to Bone Formation and Maintenance

**FIGURE 6:**
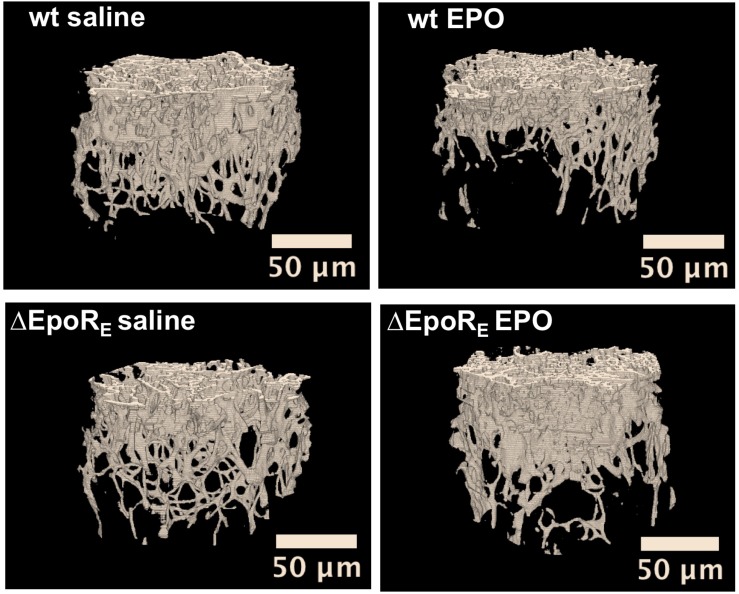
Endogenous and exogenous EPO signaling regulates bone formation. Micro-CT 3D images of trabecular bone from wild type (wt) mice and mice with EPOR restricted to erythroid tissue (ΔEpoR_E_) (Suzuki et al., 2002). Mice (age 8 weeks) were treated with EPO at 1200 U/kg for 10 days (EPO) or saline. Images from saline treatment **(left)** show reduction in bone formation in ΔEpoR_E_ mice that lack EPOR in non-hematopoietic tissue. Furthermore, the reduction in bone parameters with EPO treatment in wild type mice **(top)** is not seen in ΔEpoR_E_ mice **(bottom)**, indicating that bone loss with EPO treatment is mediated by non-erythroid response (from Suresh et al., 2019, with permission).

Understanding of the effect of EPO in bone is derived predominantly from animal models with overexpression of EPO or acute EPO administration. Recent studies using ΔEpoR_E_ mice demonstrated the importance of endogenous EPO signaling in bone formation and maintenance. These mice had significant reduction in trabecular bone in both male and female mice of mature skeletal age (Suresh et al., 2019; Figure 6). Osteoblasts and BMSCs from ΔEpoR_E_ mice did not express EPOR, while osteoclasts expressed EPOR because the erythroid transgene in this model was GATA1 dependent and preosteoclasts express GATA1, suggesting that reduction in bone formation in these mice is related to loss of EPO response in osteoblasts rather than osteoclast activity. Administration of EPO in ΔEpoR_E_ mice increased hematocrits but did not reduce trabecular bone (Figure 6) indicating that EPO induced bone reduction is independent of erythropoiesis.

In addition to reduced trabecular bone, lack of endogenous EPO-EPOR signaling increased bone marrow adipocytes leading to fatty marrow. This raised the possibility of endogenous EPO signaling regulating BMSC differentiation to either osteoblastic or adipogenic lineage.

### EPO Regulates Bone Marrow Stromal Cell Differentiation and Bone Formation

**FIGURE 7:**
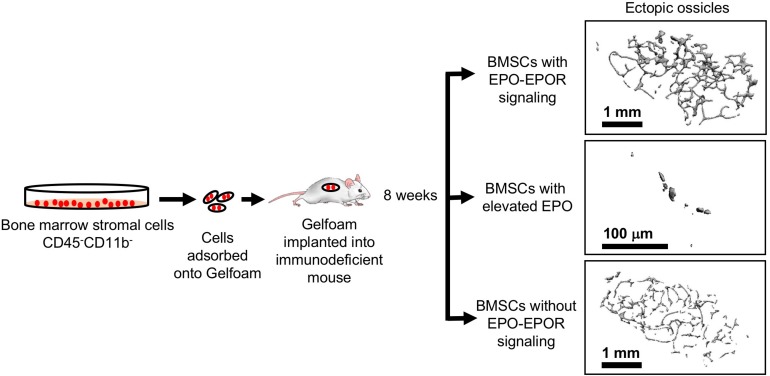
Ectopic bone formation assay with BMSC. Adherent BMSC were selected from cultures of bone marrow cells harvested from wild type and genetically altered mice. Cells were absorbed into Gelfoam and surgically transplanted into immunocompromised NSG mice (Jackson Laboratory). After 8 weeks, ectopic bone ossicles formed and were analyzed by micro-CT. Ossicles formed in transplants from ΔEpoR_E_ mice that lack EPOR in non-hematopoietic tissue **(bottom)** showed similar structure to control mice **(top)** with a trend toward fewer trabeculae, consistent with a reduction in endogenous bone formation in ΔEpoR_E_ mice. Bone formation was markedly reduced with hardly any trabecular bone in ossicles in transplants from transgenic mice expressing high level of human EPO (Ruschitzka et al., 2000) **(middle)** compared with control mice **(top)**, reflecting the even greater reduction of endogenous bone formation in these transgenic mice compared with ΔEpoR_E_ mice and control mice (modified from Suresh et al., 2019, with permissions).

The potential for BMSCs to differentiate to osteoblasts or adipocytes can be assessed using transplantation into immunodeficient mice and monitoring bone ossicle formation. *In vivo* implantation of gelatin sponge cubes (Gelfoam) carrying BMSCs in immunodeficient mice model showed that BMSCs from ΔEpoR_E_ mice that lack of endogenous EPO signaling results in reduced ectopic bone formation and increased marrow adipogenesis (Suresh et al., 2019; Figure 7). In contrast, transplanting BMSCs from transgenic mice expressing elevated EPO significantly attenuated both ectopic bone formation and marrow adiposity (Figure 7). Furthermore, systemic elevated EPO inhibited BMP2 induced ectopic bone formation without affecting osteoclasts (Suresh et al., 2019). Thus, EPO is a critical regulator of bone. In the absence of endogenous EPO signaling there is reduced bone and increased marrow adiposity. Elevated EPO is also detrimental to bone formation demonstrated by several animal models of EPO administration in healthy animals resulting in significant bone reduction. On the other hand, other animal studies point to EPO potential in accelerating bone healing. Future studies focusing on optimizing the EPO dose and duration of treatment for clinical use along with the observation of any adverse events are warranted.

## Conclusion

Hypoxia induction of EPO is an important response to ischemic stress resulting in increased red blood cell production to increase tissue oxygen delivery. Animal models have been useful in demonstrating EPOR expression beyond erythroid tissue and EPO response in non-hematopoietic tissue. For example, EPOR expression in vascular endothelium provides the potential for direct response via increase eNOS activation and NO production to regulate vascular tone and oxygen delivery (Beleslin-Cokic et al., 2004, 2011). This acute response is in contrast to the time required to stimulate survival, proliferation and differentiation of erythroid progenitor cells to produce mature red blood cells. Requirement for eNOS for EPO protective activity in heart ischemic reperfusion injury also points to the potential benefit of this acute endothelial response. EPO stimulation of improved oxygen delivery may also contribute to protective action in other non-hematopoietic tissue such as brain ischemia and skeletal muscle injury.

Erythropoietin receptor expression is not for erythroid cells only and expression in non-erythroid tissues provides for tissue specific EPO protective response during ischemic challenge or injury. EPOR expression and EPO activity include the following tissues: endothelium to regulate vascular tone, improve oxygen delivery and provide cardioprotection to ischemic injury; brain (particularly neurons) to provide protection to ischemic stress or injury; skeletal muscle (myoblasts) for tissue maintenance or repair; white adipose tissue (adipocytes and macrophages) to protect from inflammation and from increase in fat mass in male mice during diet-induced obesity; and BMSC and osteoblasts to maintain normal bone development and bone remodeling accompanying exogenous EPO stimulated erythropoiesis (Figure 1). Sites of EPO production also extend beyond fetal liver and adult kidney and include the other side of the blood-brain barrier (astrocytes and neurons) (Masuda et al., 1994; Marti et al., 1996), skeletal muscle myoblasts (Kuang et al., 2007), and osteoblasts (Rankin et al., 2012), and estrogen stimulated induction in the uterus contributing to angiogenesis (Yasuda et al., 1998), raising the possibility of EPO/EPOR autocrine response even with low EPOR expression in select non-hematopoietic cells.

In regulation of skeletal bone formation, endogenous EPO contributes to development and maintenance of skeletal bone and bone marrow adipocytes, and loss of non-hematopoietic EPO activity results in decreased bone formation and increased marrow adiposity (Suresh et al., 2019). EPO administration has also demonstrated the capacity to affect bone remodeling, but in two disparate ways: accelerate bone healing in animal models of bone fracture, and cause bone loss in healthy animals responding with increased erythropoiesis. More studies in understanding the role of EPO in bone in specific pathological conditions are warranted as patients with diseases associated with high circulating EPO such as thalassemia (Vichinsky, 1998), sickle cell disease (Sarrai et al., 2007), and polycythemia vera (Farmer et al., 2013) have debilitating bone conditions.

Sex-specific differences in plasma concentration of EPO are not detected (Jelkmann and Wiedemann, 1989). However, estrogen can affect EPO response and confer gender specific EPO action. In ventilatory response in mice, hypoxia induction of EPO modulates ventilatory response, which exhibits a sex-dimorphic behavior mediated via interaction with carotid body cells that is also sensitive to ovarian steroids (Soliz et al., 2012). The sex-dependent EPO regulation of fat mass is demonstrated with EPO treatment during high fat diet feeding that reduces fat mass gain in male mice and in female ovariectomized mice but not in female non-ovariectomized mice and not in ovariectomized mice supplemented with estradiol pellets (Zhang et al., 2017). Endogenous EPO signaling may cooperate with estrogen regulation of fat mass in female mice but mask reduction of fat mass by exogenous EPO treatment. In human, sexual dimorphic response is observed in the association between endogenous plasma EPO concentration and percent weight change per year. A negative relationship is observed in males where the higher EPO level is related to lower percent weight change, while a positive relationship is evident in females (Reinhardt et al., 2016). Elucidation of the potential cross-talk between estrogen and EPO will further the understanding of gender-specific EPO response in non-hematopoietic tissue.

Translation of animal studies that demonstrate protective effects of EPO treatment in non-hematopoietic tissues to human disease manifestations can be problematic. Neuroprotection observed with EPO treatment in animal models of ischemic stroke and brain injury and the encouraging results in the Phase I clinical trial of EPO administration for ischemic stroke (Ehrenreich et al., 2002) did not predict the adverse events associated with the combination of EPO and systemic thrombolysis therapy (Ehrenreich et al., 2009), although some benefit with EPO treatment was suggested in further subgroup analysis of patients not receiving thrombolysis therapy (Ehrenreich et al., 2011). EPO shows promise in treatment of extremely low birth weight premature infants where thrombolysis therapy is not used. EPO treatment in extremely low birth weight infants appears to be well tolerated with no excess morbidity or mortality, and, in addition to improving anemia, is associated with overall benefit in developmental assessment and cognitive development (Fauchere et al., 2008; Juul et al., 2008; Neubauer et al., 2010; Fischer et al., 2017). The potential long-term neurodevelopmental outcomes and whether benefit is a consequence of EPO stimulated erythropoiesis to improved oxygen delivery or a direct consequence of EPO response in the premature brain await further study (Fischer et al., 2017).

## Author Contributions

All authors listed have made a substantial, direct and intellectual contribution to the work, and approved it for publication.

## Conflict of Interest

The authors declare that the research was conducted in the absence of any commercial or financial relationships that could be construed as a potential conflict of interest.

## Abbreviations

ACTH: Adrenocorticotropic hormone
AKT: Protein kinase B (PKB)
ARNT: Aryl hydrocarbon nuclear translocator (HIF-1 β)
BMP: Bone morphogenetic protein
BMSC: Bone marrow stromal cells
C2C12: Mouse myoblast cell line
EGLN1: Egl-9 Family Hypoxia Inducible Factor 1 (PHD2)
eNOS: Endothelial nitric oxide synthase (NOS3)
EPAS1: Endothelial PAS Domain Protein 1 (HIF-2 α)
EPO: Erythropoietin
EPOR: Erythropoietin receptor
ERK: Extracellular signal-regulated kinase
ET-1: Endothelin-1
FGF: Fibroblast growth factor
FIH-1: Factor inhibiting HIF-1
GATA: GATA binding protein
HIF: Hypoxia-inducible factor
JAK: Janus kinase
MDS: Myelodysplastic syndrome
mEPOR −/−: Erythropoietin receptor knockout mouse
Myf-5: Myogenic factor 5
MYF6: Myogenic factor 6
MyoD: Myoblast determination protein 1
NAD: Nicotinamide adenine dinucleotide
NF- κ B: Nuclear factor kappa-light-chain-enhancer of activated B cells
NO: Nitric oxide
PAX7: Paired box 7
PGC-1: Peroxisome proliferator-activated receptor gamma coactivator 1-alpha
PHD: Prolyl hydroxylase
PI3K: Phosphoinositide 3-kinase
POMC: Proopiomelanocortin
PPAR α: Peroxisome proliferator-activated receptor alpha
PRDM16: PR-domain-containing 16
RAW264.7: Mouse macrophage cell line
Sirt1: Sirtuin 1
STAT: Signal transducers and activators of transcription
TAL1: T-cell acute lymphocytic leukemia protein 1
U/kg: Units per kilogram (EPO dose)
UCP1: Uncoupling protein 1
VHL: Von Hippel-Lindau protein
WSXWS: Amino acid motif (tryptophan-serine-X-tryptophan-serine)
Δ EpoR_E_: Mice with EPOR restricted to erythroid tissue
α-MSH: Alpha-melanocyte stimulating hormone.
